# South america’s pasture intensification can increase beef production, reduce emissions by 30% and mitigate warming from methane by 2050

**DOI:** 10.1038/s41598-025-20394-y

**Published:** 2025-10-13

**Authors:** Ciniro Costa, Luis Orlindo Tedeschi, Ricardo Gonzalez-Quintero, Jacobo Arango, Stefan Burkart, Godefroy Grosjean, Kyle M. Dittmer, Eva Wollenberg, Gonzalo Becoña, Laurent Micol, Eduardo Bustos Palma, Alejandro Ramsay Lagos, Sandra Loaiza, Marcelo Insaurralde, Luciano Guariniello, Claudia Faverin, Paulo Maria Recavarren, Maria Paz Tieri, Luiz Fernando Dias Batista, Walter Baethgen, Idupulapati M. Rao

**Affiliations:** 1 International Center for Tropical Agriculture (CIAT), Cali, Colombia; 2 Department of Animal Science, Texas A&M University, College Station, TX USA; 3 University of Vermont (UVM), Burlington, VT USA; 4 Instituto Nacional de Investigación Agropecuaria (INIA), Montevideo, Uruguay; 5 CarbonPec, Piracicaba, Brazil; 6 Gestiona, Santiago, Chile; 7 Desde El Suelo, Asunción, Paraguay; 8 Facultad de Ciencias Exactas y Naturales, Universidad Nacional de Mar del Plata, Buenos Aires, Argentina; 9 Instituto Nacional de Tecnología Agropecuaria (INTA), Balcarce, Argentina; 10 International Research Institute for Climate and Society (IRI), Columbia University, New York, NY USA

**Keywords:** Low-emissions livestock, Beef cattle production, Improved practices, Climate targets, Climate-change mitigation, Projection and prediction

## Abstract

A limited understanding of the potential to reduce emissions and a lack of climate incentives hinder progress toward mitigating greenhouse gas (GHG) emissions from beef production. This study explored the GHG mitigation potential in South America by evaluating nearly 30 beef cattle production systems across five key beef-producing countries (Argentina, Brazil, Colombia, Paraguay, and Uruguay). The study outlined a low-emission beef roadmap for this major beef producing region. Data from this study indicate that the current business-as-usual trajectory of improvements in South America’s beef cattle production is insufficient to reduce GHG emissions at a pace that aligns with the urgency of climate crisis. Results from this study show that scaling up existing practices -such as improved forages, rotational grazing, and feed supplementation- to match the performance of the region’s lowest-emission systems at 20^th^percentile could deliver significant results. Emission intensities could decrease by 33–50% compared to the projected 2050 regional average (35 tons carbon dioxide equivalent/ton of carcass weight). This would flatten the emissions curve, cutting total emissions by 20–40% while simultaneously increasing beef production by 43%. With annual methane (CH_4_) emission reductions by 1.5%, the warming effect could decrease by 70–90%, offering a transformative pathway to lower GHG emissions from beef production. This emissions trajectory offers a feasible path toward net-zero warming from beef production, primarily through sustained reductions in CH_4_ emissions intensity and absolute emissions as systems become more production efficient. These findings highlight the need and an opportunity for a drastic reduction in emissions from beef cattle production and can foster collaboration among conservation, industry, and finance stakeholders towards a common climate-oriented beef production agenda.

## Introduction

The Paris Agreement’s objective to limit global temperature increase to 1.5 °C above pre-industrial levels hinges on significant contributions from climate solutions within food systems^1–4^. The global mitigation effort especially needs to address greenhouse gas (GHG) emissions from beef cattle production. Currently, global food systems contribute approximately one-third of total GHG emissions, that is close to 20.0 gigatons of carbon dioxide equivalent per year (GtCO_2_e/year), with the beef value chain alone responsible for generating 34% (around 7.0 GtCO_2_e ) of these emissions^5–7^.

Projections indicate a 40% increase in beef production by 2050 compared to current levels^8,9^. If met through existing practices, emissions from this value chain could escalate to about 11.0 GtCO_2_e/year, negatively impacting biodiversity and water resources, and jeopardizing global climate and environmental targets^1^. However, beef production offers significant mitigation potential. Adopting existing technologies to improve global beef production efficiency could reduce emissions by 70%, lowering them from 7.3 to 2.5 GtCO_2_e/year while meeting 2050 food demands^5^. This mitigation potential is especially relevant for regions with lower productivity systems, such as South America, where over a quarter of global beef production originates^3,10,11^.

Although significant productivity improvements have happened, South America’s beef production has still relied on expanding cattle herds and pasture areas, with productivity lagging potential. Between 1990 and 2020, pasture area in South America increased by 23%, the cattle herd by 30%, and beef production by 70%^12,13^. Large areas of degraded pastures, estimated at 90 million hectares, contribute significantly to this inefficiency^13^. South America houses approximately 80% of developing countrie’s cultivated pasture area and most livestock keepers widely plant African grasses^14^. Implementing efficient pasture and animal management practices in the region could increase beef productivity to over 200 kg of beef per hectare per year, more than three times the current rate^15,16^. This increased productivity could free up 49 million hectares of land by 2030, which is crucial given the projected expansion of crop cultivation in the region^17^.

Changes in livestock production and management practices can deliver substantial GHG mitigation while improving beef productivity. Improved pasture management—through pasture restoration, the use of high-quality forage varieties, and rotational grazing—lowers emissions intensity by enhancing forage yield and nutritional quality, enabling faster liveweight gain and shorter production cycles^11,18^. Complementary measures such as supplementary feeding and genetic improvements further enhance feed efficiency and animal performance. In addition, silvopastoral and agroforestry systems can improve system resilience, sequester carbon, and contribute to further reduction in GHG emissions^18–21^.

Collectively, these strategies reduce emissions intensity and, when combined with more efficient herd management, can also lower absolute emissions. Because methane (CH_4_) is the dominant GHG emitted by pasture-based beef systems, this mitigation pathway offers a unique opportunity to neutralize its warming effect over time—bringing the sector closer to a net-zero warming trajectory^5,15^. As a short-lived climate pollutant, a sustained annual decline of approximately 0.35% in CH_4_ emissions is required to halt its additional contribution to global warming—an effect comparable to reaching net-zero carbon dioxide (CO_2_) emissions. This threshold is derived using the Global Warming Potential star (GWP*) metric, which accounts for the distinct atmospheric lifetimes and warming behaviors of CH_4_ compared to long-lived gases like CO_2_^22^. This distinction is critical, as only sustained reductions in absolute CH_4_ emissions—not intensity alone—can deliver long-term climate benefits under a warming-based accounting framework.

However, independent actions towards sustainability—through livestock, farm, and land management—can sometimes work at cross purposes. Concerning the potential negative consequences of production intensification, including the risk of increased absolute emissions despite reduced emissions intensity. More profitable livestock systems could incentivize both beef production and pasture expansion. These outcomes lead some observers to raise the question of whether the focus should shift towards reducing meat consumption via a plant-based diet instead of solely improving beef production^23^.

In this context, this study introduces a novel aspect to the topic by addressing the intersection of mitigation potential and production efficiency in South America’s beef sector. Unlike previous studies, which often focus on single countries^15,24,25^, this study is the first to collect and analyze data across multiple key countries in South America, providing a regional perspective. By quantifying their potential to simultaneously meet future production demands and significantly mitigate GHG emissions, the study fills a critical knowledge gap. To accomplish these objectives, the study conducted a review and evaluation of nearly 30 beef cattle production systems across five major beef-producing countries in South America accounting for about 90% of the beef production in South America in 2020: Argentina, Brazil, Colombia, AParaguay, and Uruguay^12^. GHG emissions intensities—measured as tCO_2_/t carcass weight (cw)—for these livestock systems were quantified using a Life Cycle Assessment (LCA) approach. Subsequently, the study estimated the productivity gap and mitigation potential based on 2050 beef production projections. Finally, to align South America’s livestock systems with global climate goals, the study explored the potential role of sustainable intensification and climate finance mechanisms, particularly carbon markets.

## Results

### Beef production systems, greenhouse gas emissions and land-use intensity

The beef cattle production systems in Argentina, Brazil, Colombia, Paraguay, and Uruguay, Colombia accounted for about 90% of the beef production in South America in 2020—representing both tropical and temperate beef production trends in the region^12^. In those five key countries, beef production systems exhibit similarities in terms of animal and pasture management, reflecting the shared environmental conditions and cattle-raising traditions across the region.

These countries primarily rely on grazing practices, where cattle are raised on grasslands and pastures. However, livestock production systems in these countries are also characterized by three levels of intensification: extensive, semi-intensive, and intensive systems (Table 1). Most grazing methods emphasize the use of native and improved grass species. Some systems employ rotational grazing. Animal feed supplementation and finishing on feedlots can complement pasture and grassland grazing (Table 1).

**Table 1:** Major characteristics of livestock production systems with different levels of intensification in South America*

Category	Extensive systems	Semi-intensive systems	Intensive systems
Production systems	Low-input systems with minimal intervention, characterized by traditional cattle breeding and long rearing cycles	Moderate-input systems focused on enhancing productivity through improved cattle breeding and a mix of pasture and animal feed supplementation	High-input systems with confinement feeding for rapid carcass weight gain, typically using feedlot systems for 100–200 days
Pasture type	Predominantly natural, nutrient-poor, unimproved pastures with low productivity	Improved pastures with grass-legume mixtures (e.g., Guinea grass and legumes) to enhance forage quality	Intensively managed planted pastures with high-yielding grass species, enhanced with fertilization, and rotational grazing to maximize forage availability and quality. Feedlots focused on total mixed rations (TMR) for energy-dense feeding
Pasture management	Minimal intervention; typically, no fertilization, lime application, or pasture renovation	Moderate inputs, including fertilization (e.g., phosphorus, NPK) and rotational grazing for better forage use	Higher use of inputs, including fertilization (e.g., phosphorus, NPK) and rotational grazing
Breed of beef cattle	Resilient breeds, often *Bos indicus* or mixed *Bos taurus*-*Bos indicus*, are suited toharsh environments	Adapted cattle breeds are selected for faster growth and higher productivity under improved management conditions	High-performance breeds (e.g., specialized beef cattle) are optimized for rapid growth and high-quality carcass production
Effect of breed	Late calving (2–3 years) with lower reproductive efficiency and extended production cycles	Enhanced reproductive performance; calving at 2 years with higher fertility rates due to better management	High reproductive efficiency and productivity, with specialized management ensuring optimal performance
Diet in calving phase	Reliance on natural pastures with little to no supplementation	Mineral and energy supplementation introduced to support reproductive performance	Nutrient-dense, energy-rich diets tailored to maximize reproductive outcomes and maintain cow health
Diet in rearing phase	Natural pastures are occasionally supplemented with minerals, leading to slow growth rates	Rotational grazing combined with mineral and energy supplements to promote faster growth	Energy-dense TMR diets to achieve rapid live weight gains, with tailored formulations for maximum growth
Diet in finishing phase	Forage-based diets with limited supplementation leading to slower weight gain	Moderate use of energy-rich supplements combined with high-quality forage for faster finishing	Energy-rich supplements combined with high-quality forage for faster finishing. High-energy TMR diets with phased increases in energy density to optimize carcass weight and quality
Animal management	Basic practices such as natural breeding, minimal health interventions, and limited parasite control	Controlled breeding seasons, routine health monitoring, targeted weaning, and efficient feed budgeting	Intensive monitoring of health and performance, high-frequency feeding, and detailed record-keeping in confinement systems
Description of scenarios	Low-input systems heavily reliant on natural resources with minimal productivity and extended production timelines	Systems with a moderate balance of input and output, integrating pasture improvements and targeted feeding practices	High-productivity systems that maximize resource use efficiency through advanced management and confinement feeding for finishing

*Expert consultations in Argentina, Brazil, Colombia, Paraguay, and Uruguay. TRM: total mixed rations.

As pasture-based intensification occurs in beef cattle production systems in South America, feeding quantity and quality improve significantly. Feed digestibility increases by up to 28.6% (from 55.0% in extensive rearing systems to 71.2% in intensive finishing systems). Stocking rates more than double, rising from 0.40 animal units per hectare per year (AU/ha/yr) in extensive systems to 2.20 AU/ha/yr in intensive finishing systems. Meat production per unit area also increases, with carcass weight gains increasing from 70 kg carcass weight per head per year (kg cw/AU/yr) in extensive systems to at least 230 kg cw/AU/yr in intensive systems (Table 2).

**Table 2:** Average livestock production, feed quality, nutrient intake, and major farming input use range for different pasture-based beef cattle production systems in South America.

	Calving		Rearing		Finishing		
	Extensive	Intensive	Extensive	Intensive	Extensive	Intensive	Intensive (Feedlot)
Stocking rate (AU ha^-1^)	0.40	0.80	0.47	1.15	0.50	2.20	-
Weight gain, kg cw head^-1^ y^-1^	75.0	82.50	82.50	107.50	70.0	230.0	275.0*
Dry Matter Intake, t DMI AU^-1^ y^-1^	3.97	4.18	3.21	4.61	4.30	6.95	4.60
Dry Matter Digestibility, %	56.40	62.50	55.00	64.50	55.00	71.20	80.00
Gross energy, MJ AU^-1^ day^-1^	200.7	211.1	162.4	232.96	217.3	351.4	230.4
Crude protein, %	8.20	10.60	8.10	11.70	8.10	13.40	16.0
Metabolizable Energy, MJ kg^-1^	8.24	9.45	8.00	9.39	8.00	10.37	13.0
Pasture productivity, tDM ha^-1^y^-1^	8.3	11.6	8.4	14.7	8.3	15.8	-
Pasture fertil., kg N-Urea ha^-1^y^-1^	0.00	50.0	0.00	50.00	0.00	200.0	-
Liming (Dolomite), kg ha^-1^ y^-1^	0.00	400.00	0.00	400.00	0.00	400.00	-
Diesel, L AU^-1^ y^-1^	0.00	0.00	0.00	5.71	0.00	28.74	295.9
Energy, L AU^-1^ y^-1^	0.00	14.50	0.00	26.27	0.00	0.00	77.46

AU ha^−1^ = Animal Units per hectare (AU = 450 kg of animal live weight); kg cw head^−1^ y^−1^ = Kilograms of carcass weight per head per year; t DMI AU^−1^ y^−1^ = Tons of Dry Matter Intake per Animal Unit per year; % DMD = Dry Matter Digestibility; MJ AU^−1^ day^−1^ = Megajoules per Animal Unit per day; % CP = Crude Protein; MJ kg^−1^ = Megajoules per kilogram (Metabolizable Energy); t DM ha^−1^ y^−1^ = Tons of Dry Matter per hectare per year; kg N-Urea ha^−1^ y^−1^ = Kilograms of Urea per hectare per year; kg ha^−1^ y^−1^ = Kilograms per hectare per year (Dolomite application). *Equivalent to a daily weight gain of approximately 1.3 kg.

Improved systems enhance nutrient intake and energy efficiency. Dry matter digestibility increases from 56.4% in extensive calving systems to 80.0% in feedlots, while metabolizable energy rises from 8.0 megajoules per kilogram (MJ kg^-1^) in extensive systems to 13.0 MJ kg^-1^ in feedlots. Crude protein content also increases, from 8.10% in extensive systems to 16.0% in feedlots, reflecting higher feed quality and nutrient availability (Table 2).

Pasture productivity increased in intensified systems, with biomass yields increasing from 8.3 tons of dry matter per hectare per year (tDM/ha/yr) in extensive systems to 15.8 tDM/ha/yr in intensive finishing systems. These improvements are supported by inputs, such as the annual application of 200 kg of urea fertilizer per hectare per year and approximately 400 kg of lime per hectare per year—inputs that are absent in extensive systems (Table 2).

However, intensified systems demand higher inputs, presenting trade-offs in resource use. Diesel consumption, for example, increases from zero in extensive systems to 28.74 L per animal unit per year (L/AU/yr) in intensive finishing systems and 295.9 L/AU/yr in feedlots (Table 2).

Overall, the transition from extensive to intensive systems in South America demonstrates the potential to significantly enhance beef production efficiency through better feed quality, higher digestibility, and increased stocking rates. However, it also underscores the importance of adopting practices that minimize resource use and environmental impacts to ensure sustainable intensification.

Assessing GHG emissions across various South America’s various beef production systems reveal a spectrum of emission profiles, shaped by their distinct approaches to cattle raising. Emissions intensity varied from 15.1 to 77.5 tCO_2_e/tcw and was predominantly influenced by CH_4_ emissions (r^2^ = 0.82). Methane accounted from 57 to 93% of total emissions (Table 3), notably attributable to animal enteric fermentation. In general, the more intensified the production system, the lower the contribution of animal emissions (especially CH_4_). However, more intensified production also increases emissions (N_2_O and CO_2_) from other farming operations, which are largely associated with pasture fertilization and feed production (Table 3).

**Table 3:** Greenhouse gas (GHG) emissions and land use intensities from different beef production in South America.

Production system	Total GHG emissions	Methane	Nitrous Oxide	Carbon dioxide	Methane %	Land use** (ha to produce 1 ton of cw per year)
	(kgCO_2_e/kg cw^-1^)					
Intensive (p)* + Feedlot	15.1	12.0	0.9	2.1	80%	4.7
Intensive (n + p) + Feedlot	20.1	15.6	0.9	3.6	78%	5.3
Intensive (p)* + Feedlot	22.1	15.2	1.0	6.0	69%	5.4
Intensive (p)* + Feedlot	22.5	13.7	6.4	2.4	61%	3.8
Semi-intensive (n + p) + Feedlot	23.0	16.6	5.1	1.3	72%	8.4
Semi-intensive (p) + Feedlot	23.0	21.4	1.5	0.1	93%	8.0
Semi-intensive (p) + Feedlot	23.2	11.4	0.9	10.9	49%	7.0
Semi-intensive (p) + Feedlot	23.8	14.6	1.1	8.1	62%	7.0
Semi-intensive (p) + Feedlot	24.1	22.5	1.5	0.1	93%	6.5
Semi-intensive (p) + Feedlot	25.0	20.5	3.6	0.9	82%	10.0
Semi-intensive (p) + Feedlot	25.1	19.1	4.8	1.1	76%	9.7
Semi-intensive (n + p)	25.4	21.0	3.7	0.7	83%	9.9
Semi-intensive (n + p)	25.9	20.3	4.6	1.0	78%	10.7
Intensive (p)	26.0	14.9	5.9	7.6	58%	4.1
Semi-intensive (p) + Feedlot	26.6	15.1	1.0	10.5	57%	10.7
Semi-intensive (p)	27.7	20.2	1.2	6.3	73%	7.0
Semi-intensive (p) + Feedlot	28.6	20.4	6.2	2.1	71%	7.0
Semi-intensive (n + p)	30.1	26.9	1.6	1.6	89%	7.2
Semi-intensive (p)	30.1	22.1	5.8	2.1	74%	7.6
Semi-intensive (n + p)	31.0	27.9	1.6	1.5	90%	7.0
Semi-intensive (n) + Feedlot	31.9	18.8	0.9	12.2	59%	9.4
Extensive (n + p)	35.8	25.5	1.7	8.7	71%	11.7
Semi-intensive (n + p)	36.5	31.4	4.3	0.8	86%	13.8
Semi-intensive (n + p)	36.5	30.6	4.9	1.0	84%	13.9
Extensive (n + p)	36.6	26.6	1.7	8.3	73%	10.4
Semi-intensive (p) + Feedlot	37.3	31.4	4.9	1.0	84%	14.1
Extensive (n)	39.7	34.9	4.2	0.6	88%	15.4
Extensive (p)	41.1	31.3	1.9	8	76%	27.1
Extensive (n)	43.4	30.8	1.7	10.9	71%	12.2
Extensive (n + p)	44.0	31.9	1.7	10.5	72%	11.0
Extensive (n)	46.4	40.9	4.9	0.6	88%	18.3
Extensive (p)	54.1	47.9	5.8	0.3	89%	19.2
Extensive (n)	54.3	46.6	2.8	4.9	86%	32.9
Extensive (n)	77.5	71.5	3.7	2.3	92%	37.9
Average	32.7	25.6	3.1	4.1	76.6	11.6
Enhanced scenario-Pctl 10^th^	22.7	14.7	0.9	0.6	59.4%	5.4
Enhanced scenario-Pctl 20^th^	23.5	15.4	1.2	0.9	70.1%	7.0
Enhanced scenario-Pctl 40^th^	26.1	20.4	1.7	1.5	73.0%	8.1
South America–2020	41.6	33.2				18.5
South America–BAU 2030	38.5	30.7				17.2
South America–BAU 2050	35.0	28.0				15.6

*Type of pasture: (p) = planted pasture, (n) = natural pasture, (p + n) = a combination of planted and natural pastures, used during one or more phases of beef production (calving, rearing, or finishing). Similar system names (e.g., “Intensive (p) + Feedlot”) may appear multiple times as they represent distinct country-specific or farm-level implementations within the same typological class. **Includes area for cropland used in feed production outside of pasturelands.

Systems in the lowest 10th percentile of emissions are shown in green, those in the 20th percentile in blue, and those in the 40th percentile in orange. Rows shaded in red indicate the emission levels of systems potentially associated with current (2020) values and projected business-as-usual (BAU) emissions for South America in 2030 and 2050.

Broadly, beef production systems that predominantly employ traditional extensive grazing tend to generate higher GHG emissions intensity (35.8–77.5 tCO_2_/t cw produced) compared to more intensive production systems that incorporate improved pastures, rotational grazing, and feed supplementation (Tables 1 and 3). These systems usually offer low animal feeding quantity and quality, which prevents animals from being finished within 24 months. Conversely, production systems that combine intensive pasture practices (e.g., pasture fertilization, feed supplementation and rotational grazing) with feedlot systems for finishing typically exhibit the lowest emissions intensity (15.1–22.5 tCO_2_/t cw produced). Meanwhile, systems incorporating semi-intensive use of inputs and interventions demonstrate an intermediate GHG emissions intensity (23.0–37.3 tCO_2_/t cw produced) (Table 3).

The variation in land-use intensity between the different systems is as notable as the variation in emissions intensity (Table 3). Extensive systems require 11.7–37.9 ha of agricultural land to produce 1 ton of carcass per year, which includes the area for cattle grazing as well as the areas for silage and crop production for cattle feed. This area decreases to 4.1–14.1 ha for the semi‒extensive systems and to 3.8–5.4 ha for the intensive systems, representing an average reduction of 63–81%, respectively (Table 3). This significant land-sparing effect could contribute indirectly to reducing emissions from deforestation and increasing removals through reforestation, as it may free up land for agricultural expansion, forestry, conservation, and restoration without the need for pasture expansion^26^. This comparative analysis underscores the importance of tailored mitigation strategies based on pasture intensification, considering the unique characteristics and challenges within each country’s beef production system^8,10,27^.

### Mitigation potential with large scale intensification of pasture-based beef production in South America

In 2020, South America produced around 14.7 million tons of beef (cw), resulting in emissions of approximately 0.67 GtCO_2_e and an emission intensity of 41.6 tCO_2_/t cw^8,12^. To meet the growing market demand, South America is expected to increase beef production by 43%, reaching 23.1 million tons by 2050^8^. The region has been steadily increasing its beef production over the last three decades^12^. Business-as-usual (BAU) productivity improvements are supposed to reduce beef production emissions intensity by only 15.9% by 2050 (to 35.0 tCO_2_e/t cw)^8,12^. GHG emission intensities from beef production for 2030 and 2050 were estimated using FAO-STAT emissions data and FAO-Outlook production projections, focusing on enteric fermentation and manure management while excluding carbon pool changes (e.g., soil carbon) and emissions from other farming production sources (e.g., energy, fertilizers, lime application, and feed production), likely to make estimates conservative.

**Fig. 1:**
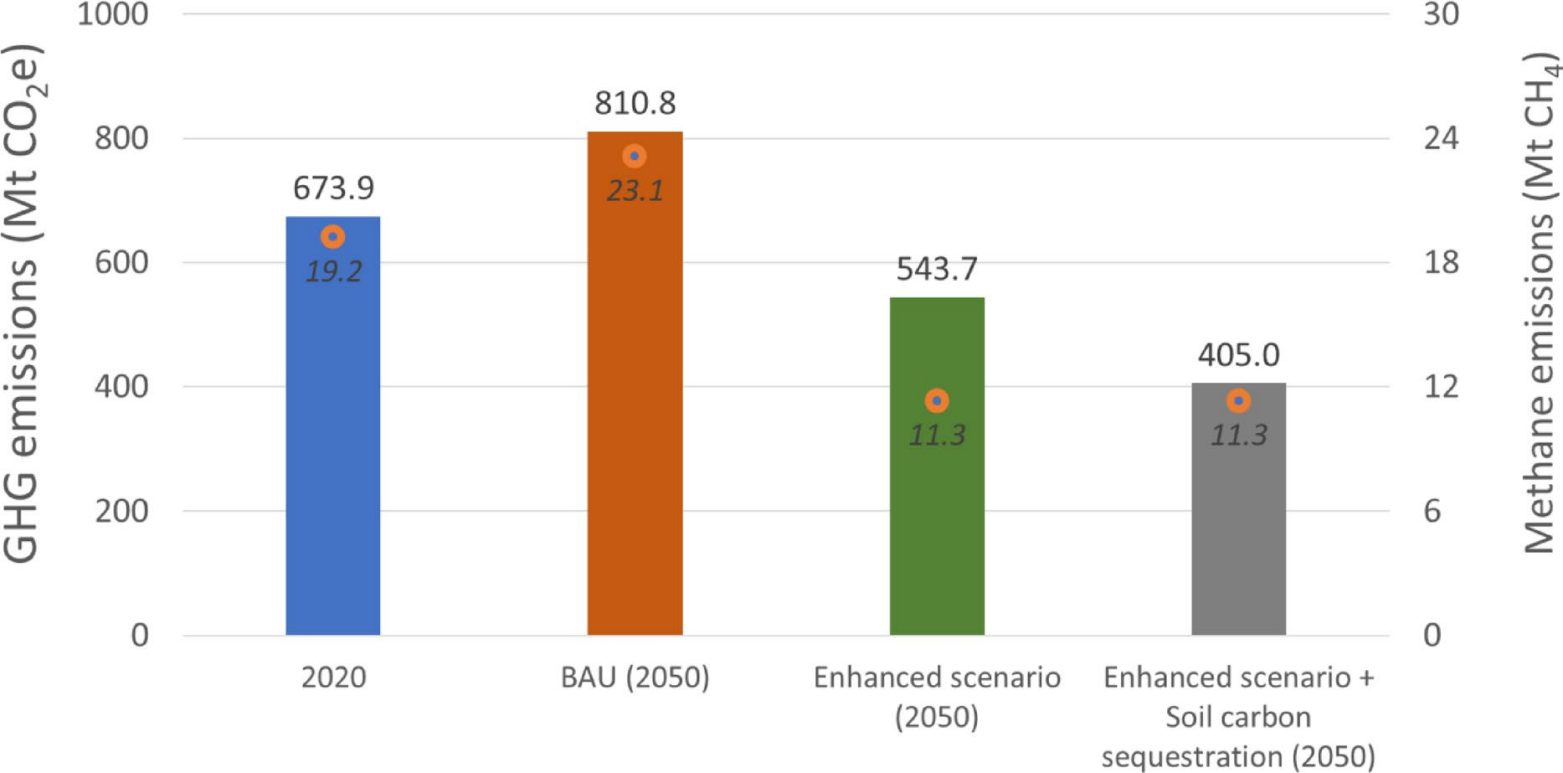
Total GHG emissions (bars, MtCO_2_e) and methane emissions (orange dots, MtCH_4_; values in italics) from South America’s beef production systems. Results are presented for the 2020 baseline (blue bar), the 2050 Business-as-Usual (BAU) projection (orange bar), and two intensified pasture-based scenarios: the Enhanced Scenario (green bar, representing the top 20th percentile of low-emitting production systems in the region) and the Enhanced Scenario + Soil Carbon Sequestration (gray bar, accounting for additional soil carbon gains from grasslands).

This level of BAU emission intensity, if maintained, falls short in reducing overall emissions. On the contrary, it would increase total GHG emissions by 20.9% (from 0.67 to 0.81 GtCO_2_e) by 2050 as the projected increase in beef production (43%) would outpace the level of mitigation (15.9%) (Fig. 1). Our study results indicate that the existing production systems in the region with emissions at the 20^th^ percentile could potentially reduce emission intensities by 33% compared to the projected regional average by 2050 (35.0 tCO_2_e/t cw) (Table 3). The emissions level of these improved systems (23.5 tCO_2_e/t cw) could flatten the emission curve, reducing total emissions in 2050 by 20% compared to 2020 levels (from 0.67 to 0.54 GtCO_2_e) while producing 43% more beef (Fig. 1).

In parallel, improving pasture-based beef production systems with lower emission intensities may also unlock carbon sequestration in soils^5^. Roe et al.^3^ estimated a cost-effective soil carbon sequestration potential in grasslands at 139 MtCO_2_e/y over 2020–2050. Emission intensity for beef production in the region may reach 17.5 tCO_2_e/t cw and reduce total emissions by 40% compared to 2020 levels (Fig. 1). Taken together, these results indicate that the mitigation potential of large-scale pasture intensification is expected to deliver around a 30% reduction in total GHG emissions by 2050 compared to 2020 levels—positioned between a ~ 20% reduction when soil carbon sequestration is excluded and a ~ 40% reduction when it is included (Fig. 1). While carbon sequestration plays an important role in the enhanced scenario, the authors recognize that its contribution may plateau over time as soils approach new equilibrium levels. In this context, additional removals beyond 2050—such as through expanding agroforestry, novel forage systems, or engineered solutions—will be needed to sustain negative emissions.^28–31^.

While all emissions in this study are calculated and reported using the GWP100 metric, it is important to consider the warming implications of decreasing CH_4_ emissions using a complementary approach. Under the GWP* framework, a sustained CH_4_ emissions reduction of approximately 0.35% per year is sufficient to halt the additional contribution of CH_4_ to global warming over a 20-year period^22,32^. In the enhanced scenario, CH_4_ emissions are projected to decline by 1.5% per year by 2050—well above this threshold. Given that CH_4_ accounts for over 50% of total GHG emissions from these systems (Table 2), the ability to substantially reduce CH_4_ emissions and neutralize its additional warming effect, hile simultaneously increasing beef production, is needed to support this scenario.

Therefore, while total GHG emissions under GWP100 remain above zero, the net warming impact of on-farm beef production in South America could be reduced by 70–90% by mid-century, primarily through CH_4_ mitigation. This would position pasture-based intensification as a key strategy for substantially lowering emissions in beef production (Fig. 1). These improvements are made possible by reductions in emissions intensity—driven by gains in feed efficiency, animal productivity, and pasture quality—as well as structural changes that limit the number of animals and pasture area needed to meet projected beef demand, resulting in lower total emissions and freeing land for other uses (Table 3).

This dual benefit—climate mitigation through lower emissions and land-use efficiency through reduced stocking rates—highlights the transformational potential of improved beef cattle production systems. These systems could reduce the pasture area required for beef production in South America by 63–81% on average, freeing up more than 130 million hectares while still meeting projected beef demand by 2050. This contrasts sharply with the BAU scenario, which would require around 20% expansion (over 60 million hectares) in pasture area to meet the same demand (Table 3).

Not all pasture areas are suitable for intensification or alternative land uses. Many extensive systems are located on degraded or nutrient-poor soils that may require substantial investment to support crop production or afforestation. However, this does not suggest that land suitability is a constraint to intensification at scale. On the contrary, our findings indicate that improved pasture-based systems can meet future beef demand using a fraction of the land required by extensive systems, while delivering greater productivity (Table 3). This land-use efficiency creates opportunities to repurpose surplus pasture area for conservation, agroforestry, or ecosystem restoration. Strategically targeting intensification in moderately productive or restorable areas—rather than in highly degraded zones—may therefore unlock the greatest potential for both climate mitigation and climate-resilient land management.

### Discussion

Intensified pasture-based beef cattle systems demonstrated lower emissions intensity compared to extensive systems. Therefore, adopting and disseminating improved production and management practices that enhance production efficiency should be the primary strategy towards reducing emissions in the South American beef industry. Effective emission intensity reduction relies on improving both animal and herd productivity rather than merely increasing productivity per unit area. In the enhanced scenarios, the primary emissions reductions were achieved through improvements in feed digestibility, feed conversion efficiency, and reduced time to slaughter—outcomes linked to production efficiency resulting from pasture improvement, supplementation, and better animal performance. These practices are currently deployable in pasture-based systems across South America, and align with several studies on sustainable beef production that prioritize pasture management and supplementary nutrition as a key first step in pasture intensification^27,33^.

For example, in the calving phase, emissions intensity is primarily influenced by the weaning rate. Better breeding practices, fixed-time artificial insemination, and effective cow management can reduce weaning rates. Enhancing pasture management and cow nutrition before breeding and during lactation further increases pregnancy rates and calf survival. Supplementary feeding for calves, or creep-feeding, also contributes to overall efficiency. Ranchers can increase productivity and reduce emissions by applying nature-based solutions, such as restoring degraded pastures and implementing rotational grazing. They can also build the necessary infrastructure for distributing supplements and water. Effective pasture management and supplementary nutrition are essential for reducing emissions intensity during rearing and finishing phases, by accelerating carcass weight gain and reducing the time animals remain in the system. Livestock producers could also adopt improvements in pasture management, including a combination of intensive pasture-based systems and feedlot systems for finishing. For optimal results, feedlot finishing should consist of a total mixed ration of 65–80% concentrate and 20–35% silage during the dry season^33,34^.

These findings align with other studies in South America^15,24,25^, which similarly highlight the relationship between management intensity and GHG emissions. For example, González-Quintero et al.^24^ and Becoña et al.^25^ observed that traditional systems with low feed quality are associated with higher emissions, whereas more intensive systems reduce emissions by improving feed conversion efficiency and animal productivity. Cardoso et al.^15^ further underscore the role of integrated practices, such as rotational grazing and targeted supplementation, in minimizing emissions and enhancing overall sustainability.

While intensification improves emissions and production efficiency, it may also require trade-offs. Extended confinement periods during feedlot for finishing can raise animal welfare concerns, and the adoption of high-performance breeds may reduce system resilience to climatic and health-related stressors compared to locally adapted breeds^35,36^. These dimensions are important to consider in scaling strategies and may influence adoption pathways in different contexts^37^.

In addition, several critical debates persist regarding intensification and its overall impact. First, enhancing pasture conditions could potentially lead to an increase in herd sizes and related emissions rather than the intended reduction. Second, the higher economic returns from intensified systems might incentivize producers to expand their pasture area, possibly promoting deforestation and offsetting the mitigation benefits of improved productions^38^. Finally, the increased beef production needed to satisfy growing demand may overshadow efforts to reduce livestock-based protein consumption as a means of cutting GHG emissions^3,4,39–43^.

However, these arguments often overlook the complex interactions between supply and demand and the pressing challenges facing beef production in the global food system. The dependence of supply on demand, as well as cost and price dynamics, must be considered. If pasture conditions and overall cattle ranching practices improve at scale, the projected demand for beef may be met with a significantly reduced land area and herd size. Conversely, if practices improve without a corresponding decrease in area and herd size, total production could exceed projected growth, raising questions about excessive supply. The hypothesis suggests that increased production will naturally lead to greater demand only if prices fall. For this to occur, either production costs or producers’ profits must decrease significantly. However, improved production methods do not substantially lower production costs, while livestock farming historically operates on low profit margins.

As intensification enhances herd productivity, enabling higher output per animal, results from this study show that it can also contribute to a gradual reduction in the total pasture area and the number of animals required over time for beef production. For instance, in the United States, beef production increased by 26% over four decades, despite a 21% reduction in the total cattle herd, demonstrating that higher output can coexist with fewer cattle^44^. South America should not replicate the U.S. model of intensive feedlots; it can continue to develop pasture-based systems that mitigate the environmental issues associated with highly intensive practices^45^.

Our findings also suggest that pasture-based intensification strategies identified in this study (Table 1)—centered on practices already available to producers, such as improved pastures, rotational grazing, and targeted feed supplementation—can deliver substantial reductions in CH_4_ emissions while improving productivity. These practices are widely adopted in the more intensive systems evaluated in this study and have been identified as primary mitigation strategies for reducing CH_4_ emissions from livestock, as they enhance forage digestibility and nutrient intake, improve feed efficiency, and shift rumen fermentation away from methanogenesis toward more energy-efficient pathways. This reduces CH_4_ emissions per unit of beef and shortens the time animals spend emitting CH_4_ during the production cycle^29^.Importantly, these practices are technically viable and compatible with the vast majority of pasture-based systems across South America, making them highly scalable and well-positioned for broader adoption. Together, they offer the most immediate and practical pathway to align the region’s livestock sector with global climate goals—particularly for CH_4_ mitigation targets outlined in initiatives such as the Global Methane Pledge^46^—and to support a broader transition toward low-emissions food systems^5^.

Importantly, the results indicate that sustained annual CH_4_ mitigation exceeding 1.5%—as projected in the improved systems scenario—surpass the ~ 0.35% threshold identified in the literature for halting additional CH_4_-driven warming under the GWP* metric. Given that CH_4_ accounts for more than 50% of total GHG emissions in many pasture-based systems, this implies that the warming impact of CH_4_ could be effectively neutralized by 2050. When combined with the land-sparing and potential carbon sequestration benefits of improved pasture management, these results point to a net-zero warming trajectory—not through complete elimination of GHG emissions, but through the strategic mitigation of CH_4_ and partial offsetting of remaining emissions via nature-based solutions.

As noted earlier, expanding agroforestry, incorporating novel forage systems, and adopting engineered solutions will be critical to maintaining long-term climate benefits. Advanced CH_4_-specific technologies such as 3-nitrooxypropanol (3-NOP) and anti-methanogenic forages (e.g., Leucaena, red algae), have demonstrated CH_4_ mitigation potential exceeding 20% in controlled trials. However, these were not included in the modeled systems due to limited field-level adoption data across the region^28,29^. Integrating improved livestock systems with crops and forestry offers another promising approach to optimize land use, where soybean and other crops are rapidly replacing pastures^47^. These integrated systems create the potential for converting some pastureland to alternative uses while intensifying cattle ranching in the remaining areas. Additionally, they can offset a portion of cattle ranching emissions through carbon accumulation in soils and trees^30,31^. For instance, silvopastoral systems in Colombia, demonstrated a negative carbon footprint of -60 kg CO_2_e per kg liveweight gain. Similar benefits can arise from native species plantations, enhancing biodiversity, resilience, and potentially increasing income^48^. Roe et al.^3^ estimate, for example, that agroforestry systems—across both livestock and non-livestock sectors—could offer a mitigation potential of up to 107 Mt CO_2_e per year in South America between 2020 and 2050. Such approaches represent viable pathways to sustain lower emissions beyond 2050, complementing improved pasture management as soil carbon sequestration begins to plateau.

However, the transition to more efficient pasture-based systems often requires significant upfront investment, which may include costs related to pasture renovation, infrastructure, and herd management. The need for substantial financial capital can hinder the adoption of intensive cattle ranching, particularly for producers with limited capital or who lack access to financing. Furthermore, extensive ranching tends to attract low-risk and low-return investors and speculators. Higher investment levels may also be less attractive due to fluctuating commodity prices.

While this study focuses on the biophysical mitigation potential of improved pasture-based systems, further research is needed to assess the financial requirements, risk-sharing mechanisms, and enabling conditions for widespread adoption. Climate finance instruments—such as carbon markets—may play a key role in supporting these transitions, particularly when designed to be inclusive of smallholder producers.

In conclusion, pasture-based intensification is not only a mitigation opportunity, but a strategic entry point for aligning the livestock sector with net-zero warming trajectories. With the right support, it can deliver meaningful emissions reductions, strengthen food security, and enhance the sustainability of beef production in South America.

## Methods

### Characterization of beef cattle production systems

This research involved a comprehensive and collaborative effort to identify and characterize major beef production systems across five target countries in South America: Brazil, Argentina, Uruguay, Colombia, and Paraguay. These systems were categorized based on farm management practices and levels of intensification, covering approximately 30 production systems ranging from extensive to intensive operations.

To achieve this, the study was conducted in partnership with renowned agricultural research institutions, including INTA in Argentina, INIA in Uruguay, EMBRAPA in Brazil, and the Alliance of Bioversity International and CIAT in Colombia, along with support from the Alliance of Bioversity and CIAT-affiliated consultants in Paraguay, among others. Local experts, including researchers, extension agents, and practitioners with in-depth knowledge of regional systems, were also integral to this process. These collaborations ensured the inclusion of region-specific insights and the accuracy of the characterization.

Experts identified three to eight common full-cycle production systems in each country, ranging from extensive to intensive operations, focusing on cow-calf, rearing and finishing operations^15,34^. Each system was assessed for its management practices, herd structure, pasture use, and other defining characteristics. This systematic approach ensured that the diversity of production systems, ranging from extensive grazing systems to highly intensive feedlots for finishing, was accurately captured.

To characterize representative production systems, data collection was conducted through structured workshops, guided interviews, and expert surveys in five countries: Brazil, Argentina, Uruguay, Colombia, and Paraguay. Five experts were interviewed in each country—including senior researchers from national agricultural research institutions (e.g., Embrapa in Brazil, INTA in Argentina, INIA in Uruguay), livestock consultants, and representatives from government agencies and the beef industry. Each consultation process was complemented by one virtual multi-stakeholder workshop per country to validate system typologies and refine technical inputs. All expert interviews, questionnaires, and workshops followed institutional guidelines by the research ethics committee of the Alliance of Bioversity International and CIAT.

A standardized questionnaire was applied across all sessions, organized around four thematic blocks: (i) System characteristics, including production typology, geographic and agroecological context, land tenure, and herd size; (ii) Livestock management, covering herd composition, breed types, reproductive strategies (e.g., calving intervals, weaning age), feeding practices by production phase, and dry matter intake; (iii) Pasture and resource use, including pasture type (natural, improved, mixed), quality, rotational grazing practices, fertilizer and lime application, diesel use, and mechanization levels; and (iv) Productivity and performance, including average daily weight gain, weaning and calving rates, final animal weights, and carcass yield.

This process generated detailed, system-specific data on animal management, forage supply, and input intensity. This harmonized dataset formed the empirical foundation for the LCA, supporting the estimation of emission factors for CH_4_ (enteric fermentation, manure), nitrous oxide (N_2_O; fertilizer application, manure), and CO_2_ (feed production, energy use, mechanization).

To facilitate cross-country comparison while preserving scientific neutrality, production systems were harmonized across countries into typologies based on shared functional characteristics—such as pasture type, input intensity, feeding strategies, and productivity levels—rather than being attributed to specific countries. This design choice reflects the intent to assess mitigation potential at the system level, independent of national boundaries. While expert consultations were conducted in each country, the resulting typologies were synthesized into regional system categories (extensive, semi-intensive, and intensive) to support technical comparability and enable cross-cutting insights. This approach avoids the disclosure of country-specific production models and ensures that the findings remain policy-relevant, technically grounded, and free from unintended commercial interpretations.

### GHG emissions metrics

All GHG emissions in this study were calculated using the GWP100 metric, following the IPCC 2006 and 2019 Guidelines for National GHG Inventories. The life cycle assessment (LCA) approach, as developed and described by González-Quintero et al.^24^, was applied consistently across cow-calf, rearing, and finishing phases in Brazil, Argentina, Uruguay, Paraguay, and Colombia. The same IPCC Tier 2 equations and input structure were used across all systems to ensure methodological consistency. Emissions were expressed in CO_2_e, allowing for comparability across CH_4_, N_2_O, and CO_2_. All *LCA results*—including emissions per hectare, per animal, and per ton of carcass weight (t/cw)—are based exclusively on GWP100 using values for CH_4_, CO_2_ and N_2_O of 28, 1 and 265, respectively^49^. The functional unit applied in this study was one ton (1,000 kg) of carcass. The LCA was done by the attributional method, which aims to quantify GHG emissions impact of a system’s main co-products in a status quo situation. Modelling of impact categories was carried out in Microsoft Excel. Calculations of GHG emissions were estimated using the 2019 Refinement to the 2006 IPCC Guidelines^50^. Table 4 summarizes the equations and emission factors (EF) used to estimate the primary emissions of CH_4_, N_2_O, and CO_2_.

**Table 4:** Emission factors (EF) for estimation of on-farm GHG emissions from cow-calf and finishing farms. Adapted from González-Quintero et al.^24^.

Pollutant	Source	Amount	References
CH_4_	Enteric	CH_4_ = [GE × (Y_m_/100) × 365)/55.65]	Equation (10.21) in IPCC^a^
		GE = [(NE_m_ + NE_a_ + NE_l_ + NE_p_/REM) + (NE_g_/REG]/(DE%/100)	Equation (10.16) in IPCC^a^
		NE_m_: net energy required by the animal for maintenance, MJ day^−1^	Equation (10.3) in IPCC^a^
		NE_a_: net energy for animal activity, MJ day^−1^	Equation (10.4) in IPCC^a^
		NE_g_: net energy needed for growth, MJ day^−1^	Equation (10.6) IPCC^a^
		NE_l_: net energy for lactation, MJ day^−1^	Equation (10.8) in IPCC^a^
		NE_p_: net energy required for pregnancy, MJ day^−1^	Equation (10.13) in IPCC^a^
		REM: the ratio of net energy available in a diet for maintenance to digestible energy	Equation (10.14) in IPCC^a^
		REG: the ratio of net energy available for growth in a diet to digestible energy consumed	Equation (10.15) in IPCC^a^
		Y_m_: 0.07 (Extensive systems)	(Gavrilova et al., 2019)
		Y_m_: 0.063 (Semi-intensive and intensive systems)	(Gavrilova et al., 2019)
		Y_m_: 0.04 (Feedlot)	(Gavrilova et al., 2019)
	Manure	CH_4_ = VS × B_0_ × 0.67 × MCF/100 × MS	Equation (10.23) in IPCC^a^
		VS = [GE × (1 − DE/100) + (UE × GE)] × [(1 − Ash)/18.45]	Equation (10.24) in IPCC^a^
		DE: feed digestibility	
		MCF: values may vary according the manure management system used	Table 10.17 in Gavrilova et al., 2019
		Bo: 0.13 (Extensive systems)	Table 10.16 in Gavrilova et al., 2019
		Bo: 0.18 (Semi-intensive, intensive, and feedlot systems)	Table 10.16 in Gavrilova et al., 2019
N_2_O-N direct	Manure and fertilizer	N_2_O-N = (F_SN_ × EF_1_) + (N_ex_ × EF_3PRP_, _CPP_)	Equation (11.1) in IPCC^a^
		EF_1_: 0.01 (0.001–0.018)	Table 11.1 in IPCC^a^
		EF_3PRP_, _CPP_: 0.004 (0–0.014)	Table 11.1 in Hergoualc’h et al. (2019)
		N_ex_ = N_intake_ − N_retention_	Equation (10.31) in IPCC^a^
		N_intake_: DMI × (CP%/100/6.25)	Equation (10.32) in IPCC^a^
		N_retention_: [(Milk × (Milk PR	Equation (10.33) in IPCCa
		%/100)/6.38] + {WG × [268 − (7.03 × Neg/WG)]/(1000 × 6.25)}	
		Milk PR%: [1.9 + 0.4 × %Fat]	
N_2_O-N indirect	Manure and fertilizer	N_2_O-N = [(F_SN_ × Frac_GASF_) + (F_PRP_ × Frac_GASM_)] × EF_4_	Equation (11.9) in IPCCa
		Frac Urea: 0.015 (0.03–0.43) Frac_GASF_ Ammonium-based: 0.08 (0.02–0.3) Frac_GASF_ Nitrate-based: 0.01 (0–0.02) Frac_GASF_ Ammonium-nitrate-based: 0.05 (0–0.2)	Table 11.3 in Hergoualc’h et al. (2019)
		Frac_GASM_: 0.21 (0.00–0.31)	Table 11.3 in Hergoualc’h et al. (2019)
		EF_4_: 0.01 (0.002–0.018)	Table 11.3 in Hergoualc’h et al. (2019)
CO_2_-C direct	Lime application	CO_2_-C = (M_Limestone_ × EF_Limestone_) + (M_Dolomite_ × EF_Dolomite_)	Equation (11.12) in IPCC^a^
		EF_Limestone_: 0.12	
		EF_Dolomite_: 0.13	
	Diesel fuel consumption	CO_2_-C = Fuel consumption × EF_Diesel_	
		EF_Diesel_: 2.23 kg CO_2_eq L^−1^ diesel^−1^	UPME (2016)

^a^The same in both the 2006 and 2019 versions. CH_4_: methane; N_2_O-N: nitrous oxide (as nitrogen); CO_2_-C: carbon dioxide (as carbon); GE: gross energy intake; Ym: methane conversion factor; NEm: net energy for maintenance; NEa: net energy for activity; NEg: net energy for growth; NEl: net energy for lactation; NEp: net energy for pregnancy; REM: ratio of net energy available for maintenance to digestible energy; REG: ratio of net energy available for growth to digestible energy; VS: volatile solids; DE: digestibility of energy (%); UE: urinary energy loss (fraction of GE); Ash: ash content in feed; MCF: methane conversion factor for manure management; Bo: maximum methane-producing capacity of manure; FSN: synthetic fertilizer nitrogen applied; EF1, EF3, EF4: emission factors for direct and indirect N_2_O; CPP/PRP: cattle and pasture rotation practices; Nex: nitrogen excretion; DMI: dry matter intake; CP%: crude protein content; Milk PR%: milk protein percentage; WG: weight gain; FracGASF: fraction of fertilizer nitrogen volatilized as NH_2_/NO_x_; FracGASM: fraction of manure nitrogen volatilized as NH_2_/NO_x_; MLimestone / MDolomite: mass of lime or dolomite applied; EFLimestone / EFDolomite: emission factors for lime/dolomite; EFDiesel: emission factor for diesel fuel.

To complement this emissions accounting framework, the GWP* (GWP star) metric was also applied in a separate analytical exercise to estimate CO_2_-warming-equivalent (CO_2_-we) values associated with CH_4_ mitigation. GWP* is a flow-based metric designed to capture the temperature impact of short-lived climate pollutants by accounting for changes in emission rates over time. Using the methodology described by Lynch et al.^32^ and Costa Jr. et al.^22^, CO_2_-e outcomes were modeled under scenarios of sustained annual CH_4_ mitigation exceeding 1.5% through 2050. These calculations were used only to conceptually illustrate the potential for reducing methane-driven warming and are clearly distinguished from the core GWP100-based LCA results.

### System boundary definition

**Fig. 2:**
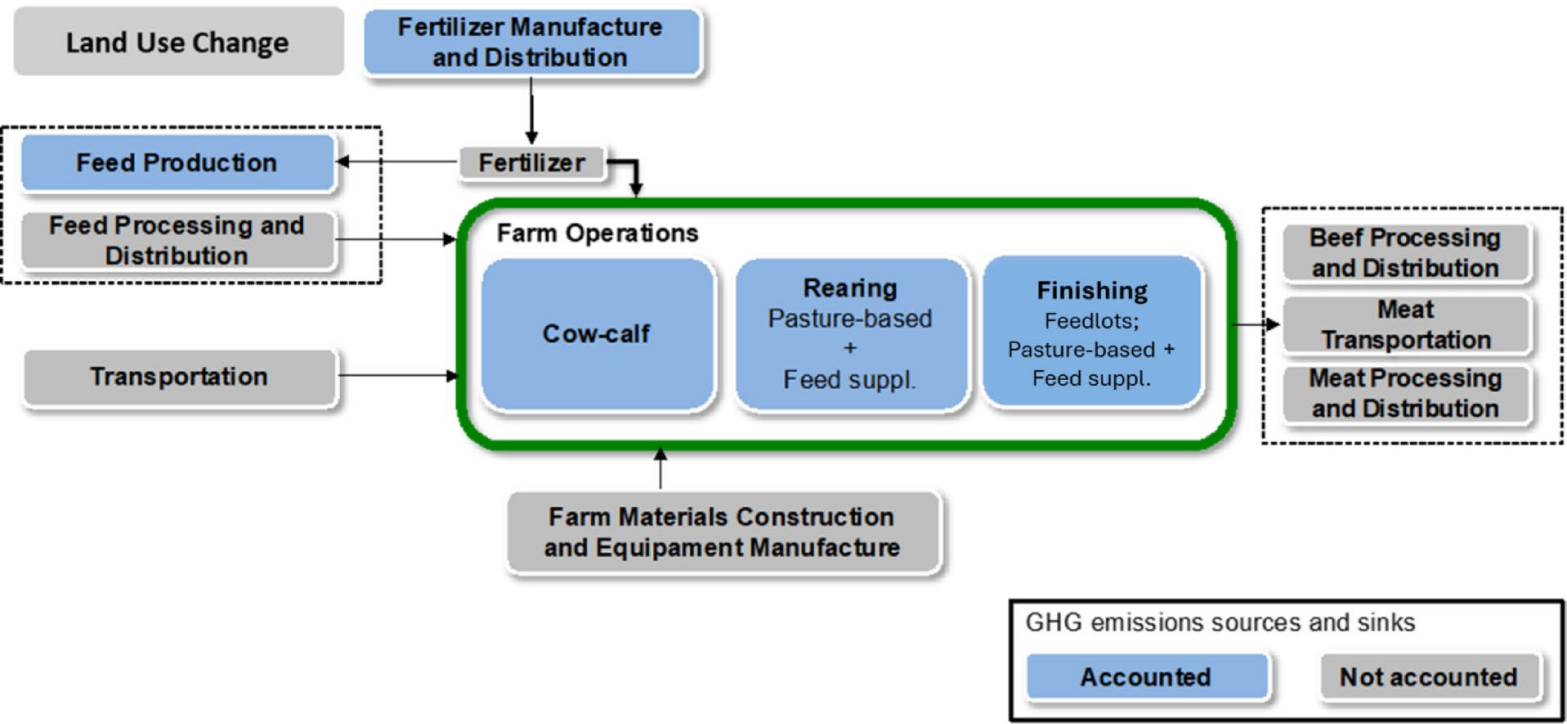
Life cycle assessment boundaries of GHG sources and sinks considered.

The system boundary was defined on a “cradle to farm-gate” perspective for cow-calf, rearing and finishing farms. The direct or primary emissions are those generated within the farm system (on-farm), and the secondary off-farm emissions are those upstream emissions related to the production and transport of imported resources such as feed, fertilizer, and soil amendments (Fig. 2). The system boundary for estimating the GHG emission from beef production includes:

- Enteric fermentation CH_4_ emissions
- CH_4_ emissions from manure management
- Direct and indirect N_2_O emissions from manure management and nitrogen fertilizer
- CO_2_ emissions from application of fertilizers (urea) and lime
- Emissions from feed production i.e., direct and indirect N_2_O emission and CO_2_ emissions from application of fertilizer, lime and crop residues.
- CO_2_ emissions from machinery used on farm and for feed production, and energy use.
- CO_2_ and N_2_O emissions from fertilizer production are needed for feed production and pasture fertilization.

### Emissions scenario development for 2050

To explore the long-term mitigation potential of pasture-based intensification, four illustrative GHG emissions scenarios were developed for the year 2050: a BAU scenario and three Improved System scenarios based on differing performance benchmarks.

For the BAU scenario, projections from FAOSTAT were used for the year 2050, which estimate CH_4_ and N_2_O emissions from beef production in South America under current trajectories. These projections account for emissions from enteric fermentation, manure management, manure left on pasture, and manure applied to soils, based on IPCC Tier 1 default methodologies and FAO activity data. The BAU scenario reflects a continuation of prevailing practices without widespread adoption of mitigation strategies.

To estimate total beef production volume in 2050, the analysis drew on projections from the FAO Agricultural Outlook 2018–2027^51^, which anticipates approximately 40% growth in beef demand in South America between 2020 and 2050. These projections served as the production baseline for all scenarios.

For the Improved System scenarios, emissions were estimated by applying the emissions intensities derived from this study across the projected 2050 beef production volume. Specifically, three scenarios were constructed based on aggregation of production systems falling within the 40th, 20th, and 10th percentiles of the emissions intensity distribution observed in the dataset. These percentiles reflect moderate, ambitious, and high-performance improvements, respectively. Each scenario assumes the region shifts toward broader adoption of existing, real-world systems already operating at those efficiency levels.

In the Enhanced Mitigation scenario, soil carbon sequestration was explicitly included in addition to improved pasture management, drawing on regionally specific estimates from Roe et al.^3^, which suggest a cost-effective sequestration potential of 139 MtCO_2_e per year from restored grasslands in South America between 2020 and 2050.

Finally, to contextualize the potential warming impact of CH_4_ mitigation, the GWP* metric was applied conceptually. Studies by Lynch et al.^32^ and Costa Jr. et al.^22^ indicate that a sustained annual reduction of ~ 0.35% in CH_4_ emissions is sufficient to halt additional methane-induced warming.

Land use intensity for 2020 was estimated by dividing beef production values by the pasture area in South America (approximately 300 million hectares)^12,13^. For the 2030 and 2050 BAU scenarios, land use intensity was estimated proportionally, based on the projected changes in emissions intensity relative to 2020 and corresponding beef production forecasts for 2030 and 2050.^8^

### Use of experimental animals and human participants

No experiments involving humans or animals were conducted in this study. All methods were carried out in accordance with the ethical standards and guidelines of the Alliance of Bioversity International and CIAT Institutional Review Board (IRB). The research consisted of expert interviews with livestock researchers, focused on professional perspectives on livestock systems, and did not involve the collection of personal or sensitive data. All participants provided informed consent prior to participation.

## Data Availability

The data supporting the findings of this study are available upon reasonable request. Researchers interested in accessing the data should contact the corresponding author. Data may be provided in formats suitable for analysis, along with relevant metadata to facilitate interpretation. To maintain the integrity of the research and protect any sensitive information, requesters may be asked to provide a clear purpose for data use and sign a data-sharing agreement, if necessary.
